# Intracranial hemorrhage in an infant leads to the diagnosis and treatment of severe hemophilia B: a case report

**DOI:** 10.1186/s13052-024-01819-2

**Published:** 2024-11-28

**Authors:** Giuseppe Lassandro, Valentina Palladino, Paola Giordano

**Affiliations:** Department of Interdisciplinary of Medicine, University of Bari ‘Aldo Moro’, Bari, 70029 Italy

**Keywords:** Case report, Coagulation factor IX, Hemophilia B, Intracranial hemorrhage, rFIX

## Abstract

**Background:**

Hemophilia B is a rare bleeding disorder in males, characterized by a deficiency in coagulation factor IX (FIX). Replacement of FIX with a recombinant FIX (rFIX) fusion protein, to sustain therapeutic plasma levels, is recommended as both treatment and prophylaxis to prevent bleeding episodes, particularly intracranial hemorrhage (ICH).

**Case presentation:**

This case report outlines the management of ICH in a 7-month-old infant with severe hemophilia B, following an accidental trauma-related event, resulting in a thin compound fracture of the left occiput. FIX levels were extremely low (˂1.0%) and large deletions of the coagulation *F9* gene (including exons 1–6) were identified. Intensive treatment with a rFIX fusion protein conjugated to the immunoglobulin Fc fragment (rFIXFc) continued for 18 days before hospital discharge. A continuous regimen of weekly rFIXFc infusions was implemented. Thirty days after initiating rFIXFc therapy, neutralizing antibodies or FIX inhibitors (common in patients with large *F9* gene deletions) were observed, causing a diffuse skin rash. Such allergic reactions typically indicate progression to potentially serious nephrotic syndrome. A unique immunotolerance regimen of oral oxatomide and intravenous hydrocortisone was started to proactively prevent allergic reactions in this patient during rFIXFc prophylaxis. Even though low titers of the inhibitor (0.6–1.0 Bethesda units) were observed occasionally during subsequent follow-up, there were no signs of further allergies or development of nephrotic syndrome.

**Conclusion:**

This is an uncommon case in which rFIXFc was continued despite the appearance of an allergic reaction and the development of FIX inhibitors. Subsequent allergic reactions were prevented with a combination of oral oxatomide and intravenous hydrocortisone given prior to prophylactic rFIXFc. Further studies are recommended to determine the usefulness of this combination with rFIX therapy.

## Background

Hemophilia B is a rare, recessive X-chromosome-linked bleeding disorder, affecting almost one in 25,000 males worldwide [1]. In patients with hemophilia B, a mutation in chromosome Xq27.1 results in reduced factor IX (FIX) activity [2]. In general, patients are managed according to disease severity (classified as mild, moderate, or severe according to factor activity level), treatment type, bleeding episodes, adverse events, joint and inhibitor status, comorbidities, and quality of life [2].

The standard treatment for hemophilia B consists of exogenous FIX replacement therapy that aims to replace and elevate plasma FIX levels [2]. Current therapeutic management in prophylactic, on-demand, or perioperative settings includes administering intravenous FIX concentrates, such as plasma-derived FIX (pdFIX) or recombinant FIX (rFIX). The pdFIX and rFIX conventional products require relatively frequent infusions (2 or 3 times a week) to maintain protective FIX levels [3]. To reduce patient burden, FIX products with 3- to 5-fold longer half-lives were developed [3], including the rFIX conjugated to the fragment crystallizable (Fc) region of human immunoglobulin G (rFIXFc) or to recombinant human albumin (rIX-FP) [4, 5]. Benefits associated with using these extended–half-life (EHL) rFIX products include a reduced frequency of injections thus promoting treatment adherence, a reduced need for central venous lines in pediatric patients, and a potential improvement in patient quality of life [3]. Several clinical studies support the prophylactic use of rIX-FP and rFIXFc in pediatric patients with hemophilia B [6–10].

According to the World Federation of Hemophilia (WFH) guidelines on the management of hemophilia, prophylactic treatment is endorsed as the standard of care for severe and non-severe hemophilia B; the rationale is to prevent bleeding episodes, as well as providing superior benefits to on-demand therapy [2]. These guidelines recommend early initiation of prophylaxis (ideally before the age of 3 years) with rFIX, EHL rFIX or other hemostatic agents, prior to joint disease onset [2] Indeed, the implementation of individualized prophylactic treatment in children has prevented progression to severe disease with lifelong disabilities [11]. Despite advances in the treatment of hemophilia with clotting factors, the development of neutralizing antibodies that inhibit their function (inhibitors) remains a major challenge, and is considered the most significant complication of treatment [12]. The highest risk of inhibitor development occurs within the first 50 exposure days of treatment initiation [3]. Immune tolerance induction (ITI) to eradicate inhibitors has been used successfully in patients with hemophilia A, but there is limited clinical experience of this strategy in patients with hemophilia B, especially given that inhibitor prevalence is low in the latter population [2].

In newborns and children less than 2 years of age with severe hemophilia, common types of bleeding include soft tissue and intramuscular bleeding and bleeding associated with a medical procedure (e.g., venipuncture, central line placement, circumcision, neonatal heel prick, oral or nasal mucocutaneous bleeding or extracranial bleeding) [2]. However, intracranial hemorrhage (ICH) is the most complex bleeding event in patients with hemophilia across all ages, and may lead to disability, or in some cases, to death [13]. The frequency of ICH in male neonates and toddlers aged 0–2 years with hemophilia B was about 8% (*n* = 6/73) in a study that collected data on the body site of the first bleed [14]. In a different study, the incidence of death due to ICH among pediatric patients with hemophilia A or B who experienced an ICH was 6.7% (*n* = 1/15) [13]. ICH can be spontaneous (more prevalent in adults) or trauma-related (more prevalent in children); the risk factors include the type of bleeding disorder and its severity, genotype and genetic polymorphisms, type of birth, and sporting activities [15]. ICH morbidity and mortality can be reduced if medical professionals, particularly in Emergency Departments (ED), are familiar with all the risk factors for ICH [15]. This case report describes the management of trauma-related ICH in an infant subsequently diagnosed with severe hemophilia B, as well as the follow-up prophylactic treatment for hemophilia.

## Case presentation

A 7-month-old male infant presented to the ED for urgent care with “uncontrollable crying, forced position and crying in position changes”. The parents reported the infant had an accidental fall from his bed 3 days prior, without loss of consciousness or vomiting. The head was normal in shape with the anterior fontanelle normally taut; his hydration and nutrition status, and cardiorespiratory and abdominal examinations were normal. There was no evidence of hemorrhages and/or bleeding.

A pathological family anamnesis (collected despite language communication difficulties) determined that the patient was born at term from spontaneous delivery at 38 weeks of gestational age (birth weight 2500 g), following a normal pregnancy. He was hospitalized for fever at the age of 6 months and treated with antipyretic drugs and antibiotics. His maternal uncle, maternal grandfather, and maternal cousin were also affected by severe hemophilia B, with large deletions of the coagulation *F9* gene that included exons 1 to 6. FIX inhibitors were noted in the maternal uncle (following prophylaxis with activated prothrombin complex) and in the maternal cousin (anaphylactic shock after rFIX). Given the patient’s family history, the patient underwent genetic testing a few weeks after being hospitalized, where deletion of exons 1 to 6 of the *F9* gene was identified.

A cranial computed tomography (CT) scan performed at admission identified mucous material completely occupying the mastoid cells and the right middle ear and a thin compound fracture of the left occiput; this was confirmed by a magnetic resonance imaging (MRI) scan. The ventricular system was not dilated and there were no densitometric alterations in the encephalic nerve tissues. Hyperdensity of the main intracranial venous sinuses was noted and hyperdense material occupied the vertebral canal down to cervical vertebra number 4, displacing the anterior marrow. Administration of contrast medium revealed no new or different results in the MRI scans. A heterogeneous formation was evident in the extramedullary site (from the cervical to the sacral tract) posterolateral to the medulla. A predominantly iso-intense signal on T1-weighted images and iso-hyperintense signal in long repetition time sequences were observed without significant contrast enhancement. This was compatible with the initial hypothesis of an epidural hematoma in the acute phase. Anterior spinal cord compression was also observed, particularly at the dorsal level.

The patient was also evaluated by a neurologist and neurosurgeon in the ED, both of which excluded the need for neurosurgical interventions and recommended referral to the specialized pediatric hematology clinic.

Laboratory test results of blood drawn in the ED at presentation revealed several abnormalities (Table 1).

**Table 1:** Laboratory test results and interpretation

	Normal range	Patient’s levels	Interpretation
PT ratio	˂1.1 [16]	1.2	Slightly high
PTT ratio	1.5–2.5 [17]	3.94	High
PT mixing ratio	1:1	1:1	
PTT mixing	1.11	1.11	After mixing, the PTT ratio normalized
Factor VIII	50–150% [2]	135%	Normal
Factor IX	50–150% [2]	˂1%	Extremely low
Hemoglobin (mean)	12 g/dL [18]	7.5 g/dL	Low

*PT* prothrombin time, *PTT* Partial thromboplastin time

Both the prothrombin time (PT) and partial thromboplastin time (PTT) were elevated. When interpreting the PTT mixing results, the correction of the PTT from 3.94 to 1.11 indicated a possible factor deficiency [19]. While Factor VIII levels were within the normal range, FIX levels were extremely low (˂1.0%), confirming a diagnosis of severe hemophilia B.

### Treatment and outcomes

The patient was hospitalized and intensive therapy with rFIXFc was initiated immediately after diagnosis and administered at a dose of 70 UI/kg. Administration at the same dose continued every 12 h to replenish plasma FIX. A transfusion of concentrated red blood cells after administration of rFIX led to rising hemoglobin levels at subsequent blood chemistry checks. The patient was also prescribed broad-spectrum oral antibiotics and analgesic therapy.

During hospitalization, the patient underwent close clinical and laboratory monitoring, as well as two follow-up MRIs. Results of these MRIs showed progressive improvement in the patient’s fracture and hemorrhaging. During hospitalization, FIX values greater than 30.0% were maintained. Intensive in-hospital rFIXFc therapy lasted 18 days and primary prophylaxis with rFIXFc was initiated prior to discharge. Close clinical monitoring was provided after discharge, with the first follow-up appointment 2 days after discharge.

One infusion of rFIXFc was administered weekly as prophylaxis at the pediatric hematology clinic. These weekly clinic visits were also used to conduct follow-up monitoring. During follow-up, the patient presented with a few transient bruises, confirming treatment efficacy. Thirty days after the initiation of intensive in-hospital rFIXFc, a contextual diffuse skin rash had appeared and a low-titer inhibitor level (0.6–1.0 Bethesda units [BU]) was detected. Intravenous hydrocortisone and oral oxatomide were added to the regimen as immune tolerance induction (ITI) therapy, and were administered prior to rFIXFc. Thereafter, only transient post-traumatic cutaneous hemorrhages appeared; therapeutic efficacy was inferred by the absence of articular hemorrhages. Although low-titer inhibitor levels (0.6–1.0 BU) occasionally appeared, there were no signs of allergies or nephrotic syndrome during follow-up. The effectiveness of the corticosteroid and antihistamine combination in achieving immune tolerance was confirmed by the absence of new skin manifestations. Inhibitor levels were checked monthly, and titers were always low (i.e., 0.6–1.0 BU). At the most recent follow-up (April 2023), the patient was still continuing treatment with the same regimen.

## Discussion and conclusions

This patient was managed in accordance with the WFH guidelines, which recommend clotting factor concentrates for treating or preventing bleeding in patients with hemophilia B, and in particular, early initiation of prophylactic therapy [2]. The FIX concentrate selected for the patient reported here was rFIXFc, which is an EHL FIX replacement therapy, and as such, plays a protective role in modulating the immune response and preventing the appearance of inhibitors [20]. While the WFH guidelines make no specific recommendations regarding the use of one type of clotting factor concentrate over another in pediatric patients, it should be acknowledged that rFIX and EHL-FIX have significantly improved the management of hemophilia B in the last 30 years [21].

A significant complication following rFIX treatment is the development of rFIX inhibitors, though this occurs only in 3.0% of patients with hemophilia B [12]. Inhibitors are primarily seen in the subgroup of patients with large deletions of the *F9* gene who typically present with severe hemophilia [22, 23], as was seen in this case. Since the formation of inhibitors is known to diminish or nullify the efficacy of FIX therapy against severe hemophilia B, the control of bleeding becomes challenging [24]. Indeed, recurrent bleeding may progress to hemophilic arthropathy [21]. The presence of rFIX inhibitors is known to lead to severe allergic reactions (e.g., anaphylactic shock) and nephrotic syndrome [2]. Importantly, the development of nephrotic syndrome can be predicted by allergic reactions to FIX concentrates [25], which was of particular concern with the infant in this case study. Though the mechanisms of allergic reactions and nephrotic syndrome are not well understood, complete gene deletions tend to significantly increase the risks of these adverse events [22].

One strategy to minimize the development of FIX inhibitors, or to eradicate them once present, is ITI therapy, whereby FIX concentrates are regularly administered with immunosuppressants in various combinations to achieve immune tolerance [22]. Typical agents used in ITI therapy include rituximab [24], cyclosporin A [25], prednisolone, dexamethasone, mycophenolate mofetil or hydrocortisone [22]. However, there are limited data for the use of ITI in pediatric patients with hemophilia B, and there remains a need for international collaborative efforts to develop standardized ITI therapy guidelines and recommendations. Nevertheless, our report illustrates that immune tolerance can be achieved in pediatric patients with hemophilia B in real-world practice, despite the possibility of failure and complications, and supports results from several previous case reports and small, observational studies in pediatric patients with hemophilia B [22, 26–28]. For example, one study documented the successful use of hydrocortisone with FIX concentrates in three patients whose FIX treatment had been previously suspended. Subsequent to the introduction of hydrocortisone treatment, no occurrences of allergic reactions, nephrotic syndrome, or serious infections were observed [28].

The WFH guidelines recommend the use of antihistamines immediately prior to infusion of clotting factor concentrates (including rFIXFc) in patients who experience repeated allergic reactions [2]. One prior report of ITI that included rituximab in the management of an adolescent with severe hemophilia B described the use of prophylactic antihistamines to manage respiratory allergic reactions that had accompanied inhibitor development during PCC treatment; however, this strategy was not successful, and the patient developed nephrotic syndrome [25]. Allergic reactions were managed in our patient by the addition of oxatomide to hydrocortisone as ITI therapy prior to administration of prophylactic rFIXFc. This combination was effective in preventing allergic cutaneous reactions or the development of nephrotic syndrome. Despite low titers of the inhibitor (0.6–1.0 BU) being occasionally detected during follow-up, this treatment regimen was well-tolerated and continues successfully.

## Limitations

A number of limitations to this case report should be acknowledged. Since data are only for a single patient, the treatment strategy and results observed in this patient may not be generalizable to all pediatric patients with hemophilia B. By its nature, a case report is retrospective, and as such may be subject to errors or omissions in the patient’s medical records, and some of the data collected relied on caregiver/guardian recall and so may be subject to recall bias.

To the best of our knowledge, this is the only case described in literature in which an infant was successfully treated with an antihistamine and corticosteroid combination, allowing for the continuation of prophylactic rFIXFc therapy, despite the appearance of a FIX inhibitor. Although FIX replacement therapy is usually contraindicated in patients who develop inhibitors [28], we continued with rFIXFc prophylaxis because we successfully prevented allergic reactions by pre-treatment with oral oxatomide and intravenous hydrocortisone. We recommend that pre-medication with antihistamines and/or corticosteroids as ITI therapy may be initiated when the early signs of an allergic reaction develops in similar cases with Hemophilia B. Further studies are recommended to determine the usefulness of antihistamines and hydrocortisone as ITI therapy to prevent allergies and avoid the development of nephrotic syndrome in pediatric patients with severe hemophilia B treated with rFIXFc.

## Data Availability

The datasets generated and/or analyzed during the current study are not publicly available due to patient confidentiality.

## Abbreviations

BU: Bethesda unit
CT: Computed tomography
ED: Emergency Department
EHL: Extended half-life
FIX: Factor IX
ICH: Intracranial hemorrhage
INR: International normalized ratio
ITI: Immune tolerance induction
MRI: Magnetic resonance imaging
pdFIX: Plasma-derived FIX
PCC: Prothrombin Complex Concentrate
PT: Prothrombin time
PTT: Partial thromboplastin time
rFIX: Recombinant Factor IX
rFIXFc: Recombinant Factor IX fusion protein conjugated to immunoglobulin Fc fragment
WFH: World Federation of Hemophilia
